# Complete Genome Sequence of Bacillus megaterium Myophage Mater

**DOI:** 10.1128/genomeA.01424-14

**Published:** 2015-01-15

**Authors:** Jacob C. Lancaster, Mary K. Hodde, Adriana C. Hernandez, Gabriel F. Kuty Everett

**Affiliations:** Center for Phage Technology, Texas A&M University, College Station, Texas, USA

## Abstract

Bacillus megaterium is a ubiquitous, soil inhabiting Gram-positive bacterium that is a common model organism and is used in industrial applications for protein production. The following reports the complete sequencing and annotation of the genome of B. megaterium myophage Mater and describes the major features identified.

## GENOME ANNOUNCEMENT

Bacillus megaterium is a non-pathogenic bacterium that is the largest of all bacilli (1). It is commonly used as a model system for a variety of cellular processes including morphology, DNA partitioning, and sporulation. B. megaterium also serves as a host for commercial production of a wide range of biologics including vitamin B_12_ and amylases (2). It was also classically used to study lysogeny of bacteriophage (3). Here, we present the genome of myophage Mater, which was isolated on asporogenic B. megaterium KM Sp^-^.

Bacteriophage Mater was isolated from a soil sample collected Huntsville, TX, USA. Phage DNA was sequenced in an Illumina MiSeq 250-bp paired-end run with a 550-bp insert library at the Genomic Sequencing and Analysis Facility at the University of Texas (Austin, TX, USA). Quality controlled, trimmed reads were assembled to a single contig at 53.2 fold coverage using Velvet version 1.2.10. Contigs were confirmed to be complete by PCR. Genes were predicted using GeneMarkS (4) and corrected using software tools available on the Center for Phage Technology (CPT) Galaxy instance (https://cpt.tamu.edu/galaxy-public/). Phage morphology was determined by transmission electron microscopy performed at the Microscopy and Imaging Center at Texas A&M University.

Phage Mater has an icosahedral head containing a 164,302-bp genome. The unit genome has a G+C content of 39.52%, a coding density of 91%, and encodes 6 tRNA genes. A 9,227 bp long-terminal repeat was identified using the PAUSE method (https://cpt.tamu.edu/pause/) on raw sequencing data. It has 222 predicted unique coding sequences, of which 72 are hypothetical novel, 93 are hypothetical conserved, and 57 have an annotated gene function. Mater has a limited host range and also infects B. megaterium strain PV361.

Mater is similar to Bacillus phages phiNIT1 and Grass (NC_021856 [50.5% identity] and NC_022771 [50.0% identity], respectively) as determined by Emboss Stretcher (5). Using BLASTp and InterPro Scan analyses, genes encoding proteins related to a variety of functions (morphogenesis, replication/recombination, biosynthesis, gene regulation, and lysis) were annotated (6, 7). Several morphogenesis proteins including the portal protein, prohead protease, tail fiber, spike, tail lysin, sheath, and major capsid protein were identified, but genes encoding other major myophage structural proteins were not found, presumably due to sequence divergence (8). Replication and recombination genes (DNA-binding proteins, DNA polymerase, primase, Holliday junction resolvase, RecA, and helicase) were readily annotated by sequence homology. In terms of lysis genes, a class-II holin was found (containing two transmembrane domains in an N-in, C-in topology) as well as three endolysin candidates: a peptidoglycan-binding peptidase, an *N*-acetylmuramoyl-l-alanine amidase, and a membrane-bound glycoside hydrolase.

Mater has many interesting features including an RtcB-like tRNA ligase and an FtsK/SpoIIIE DNA pump. In Escherichia coli, the RtcB tRNA ligase repairs tRNA molecules damaged by cellular stress (9). In Gram-positive bacteria, SpoIIIE acts as a pump to move DNA into the forespore during sporulation (10). The role these proteins play in the phage infection cycle remains undefined.

### Nucleotide sequence accession number.

The genome sequence of phage Mater was contributed as accession no. KM236245 to GenBank.
